# Supporting data for strengthening and deformation behavior of as-cast CoCrCu_1.5_MnNi high entropy alloy with micro-/nanoscale precipitation

**DOI:** 10.1016/j.dib.2022.108567

**Published:** 2022-09-01

**Authors:** Sang Hun Shim, Hesam Pouraliakbar, Byung Ju Lee, Yong Keun Kim, Mohsen Saboktakin Rizi, Jun Hyun Han, Sun Ig Hong

**Affiliations:** Department of Materials Science and Engineering, Chungnam National University, Daejeon 34134, Republic of Korea

**Keywords:** As-cast strength, Strength/ductility, Gage length to width ratio, High entropy alloy (HEA), Precipitate strengthening, Diffraction intensity line profile

## Abstract

The data presented here are related to the research article entitled “Strengthening and deformation behavior of as-cast CoCrCu_1.5_MnNi high-entropy alloy (HEA) with micro-/nanoscale precipitation [1]”. Non-equimolar CoCrCu_1.5_MnNi was cast by the conventional induction melting under a high-purity Ar atmosphere. Scanning electron microscopy equipped with energy dispersive spectroscopy (EDS), and transmission electron microscopy (TEM) were used for micro- and nanostructure characterization. Subsize tensile specimens with two different gage length to width ratio were tested at room and cryogenic temperatures to assess the accuracy of strength and ductility data in the as-cast CoCrCu_1.5_MnNi HEAs. The mixing enthalpy (ΔH_mix_) versus lattice elastic energy (ΔH_el_) criterion was used to predict the stable phases. The data on the effects of microstructural and nanostructural distribution of various phases on mechani-cal properties in the as-cast HEA could be used in designing high entropy alloys with excellent as-cast mechanical performance.

**Specifications table**

**Table:** 

Subject	Metals and alloys
Specific subject area	Mechanical performance and microstructural evolution of high-entropy alloys (HEAs)
Type of data	Table (Phase fraction of matrix and precipitates, chemical composition of phases, Calculated mixing enthalpy (ΔH_mix_), lattice elastic energy (ΔH_el_)). Figure (Mechanical properties, Thermodynamic data, SAED patterns and SAED line scan, SEM and EDS, Precipitate strengthening calculation)
How data were acquired	Scanning electron microscopy (SEM), energy dispersive spectroscopy (EDS), transmission electron microscopy (TEM), mechanical testing.
Data format	Raw data (SEM, EDS, TEM images, Stress strain curves). Analysed and measured data (Thermodynamic analyses, lattice constants, size and distribution of precipitates), Analysed: Thermodynamic criterion and precipitate strengthening calculation.
Parameters for data collection	Sub-size tensile specimens with different gage length to width (GLW) ratio (effect of GLW ratio on the mechanical properties). Testing temperatures (room temperature and 77 K). Transmission-electron microscopy (TEM) analyses were carried out employing JEOL JEM-2100F operated at an acceleration voltage of 200 kV.
Description of data collection	Metallography samples were prepared by cutting, grinding, and mechanical polishing. TEM foils of as-cast CoCrCu_1.5_MnNi were prepared using a dimple grinder (Gatan, model 656, USA) and the precision ion polishing system (Gatan, model 691, USA), with argon gas ions at 3.6 keV. Tensile stress-strain curves at RT and 77 K.
Data source location	Institution: Chungnam National University City/Town/Region: Daejeon Country: Republic of Korea
Data accessibility	Data are with the article. The raw data are in the Mendeley Data repository. https://data.mendeley.com/datasets/thbg743tdz/2
Related research article	Sang Hun Shim, Hesam Pouraliakbar, Byung Ju Lee, Yong Keun Kim, Mohsen Saboktakin Rizi, Sun Ig Hong, Strengthening and deformation behavior of as-cast CoCrCu1.5MnNi high entropy alloy with micro-/nanoscale precipitation, Materials Science and Engineering: A. 853 (2022) 143729 [1], https://doi.org/10.1016/j.msea.2022.143729.

## Value of the Data

• The datasets on the effect of gage length to width ratio on the stress-strain curves can be used to understand the influence of gage length to width ratio on the strength, ductility in the sub-size tensile specimens in CoCrCu_1.5_MnNi HEA and other engineering alloys.
• The data on the phase separation and phase distribution in the dual fcc phase structure of CoCrCu_1.5_MnNi would help the researchers to understand the effects of phase distributions on the mechanical properties and provide the design strategy for as-cast high entropy alloys with improved mechanical performances.
• The thermodynamic calculation data on the stepwise phase separation of as-cast non-equiatomic CoCrCu_1.5_MnNi high entropy alloy with dual fcc phase structure can also be used to provide the database for the design of high entropy alloys with precipitation in the as-cast structure.

## 1 Data Description

**Fig. 1:**
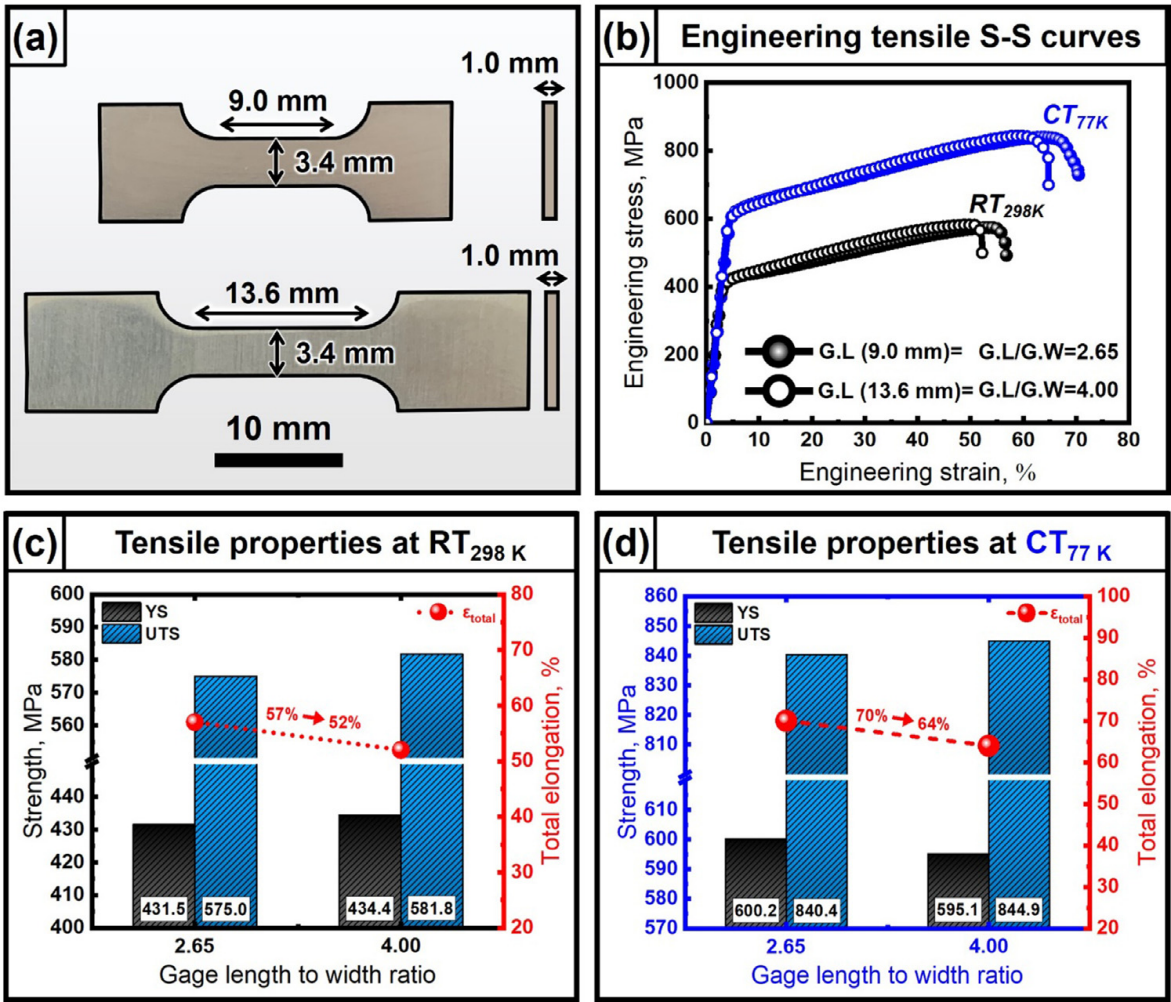
(a) A plan view of the tensile specimen with gage length (G.L) of 9 and 13.6 mm, (b) the engineering stress-strain curves of as-cast CoCrCu_1.5_MnNi HEA with different gage length at room and cryogenic temperatures. Summarized tensile properties (c, d) with gage length to width ratio at room (c) and cryogenic (d) temperatures.

In this article, the data on the nanostrucure, mechaical properties and the thermodynamic criterion for phase stability of as-cast non-equiatomic CoCrCu_1.5_MnNi high entropy alloy [1] are provided. The data on the accuracy of mechanical testing using the subsize specimens are also provided. Sergueeva et al. [2] reported the effect of stress-strain behaviors for specimens with different gage length (GL)/gage width (GW) ratio on the various materials. With the decrease of the gage length to width ratio, GL/GW, the calculated ductility increases because of the localized deformation observed in most engineering alloys and the reduced gage length. To ensure the accurate measurements of the elongation, it has been suggested that the optimized GL/GW ratio is recommend to be 4.0, by the ASTM standard E8 (American Society for Testing and Materials) [3]. Fig. 1(a) shows the plan view of the dogbone-type tensile specimens with the gage length of 9mm or13.6mm and the width/thickness of 3.4mm/1.0mm. Fig. 1(b) exhibits the engineering stress-strain curves for the specimens with the gage length of 9 and 13.6 mm (GL/GW ratio of 2.65 and 4.0, respectively) at both 298 K and 77 K, for the as-cast CoCrCu_1.5_MnNi HEA. The raw data of engineering stress-strain curves are stored in the Mendeley Data repository. It is shown that the specimens with a larger gage length ratio (4.0) have the decreased ductility than the specimens with a smaller gage length of 2.65 both at 298 K and 77 K as shown in Fig. 1, At 77 K, the mechanical properties, such as strength and total elongation were enhanced. The decrease of the ductility with the increase of the gage length to width ration was found to be larger at 77K than that at 298K. The yield strength and ultimate tensile strength were found to be unaffected by the variation of the gage length to width ratio, but the ductility was found to (∼3% at 298 K and ∼6% at 77 K) decrease slightly with the increase of gage length to width ratio from 2.65 to 4 in CoCrCu_1.5_MnNi HEA. Since the ductility is defined as ΔL_f_/ L_i_, i.e., the difference (ΔL_f_) of the elongated gage length at fracture (L_f_) and the initial gage length (L_i_) divided by the initial gage length (L_i_), the measured ductility tend to increase with decrease of the initial gage length (L_i_) because the strain tends to be localized in the central region of the gage length [1]. The effects of the gage length to width ratio on the stress strain curves, the ductility and the strength at 298 K and 77 K are exhibited in Fig. 1(c, d). The strength and ductility data in Fig. 1(c) and (d) are summarized and stored in Mendeley Data repository.

**Fig. 2:**
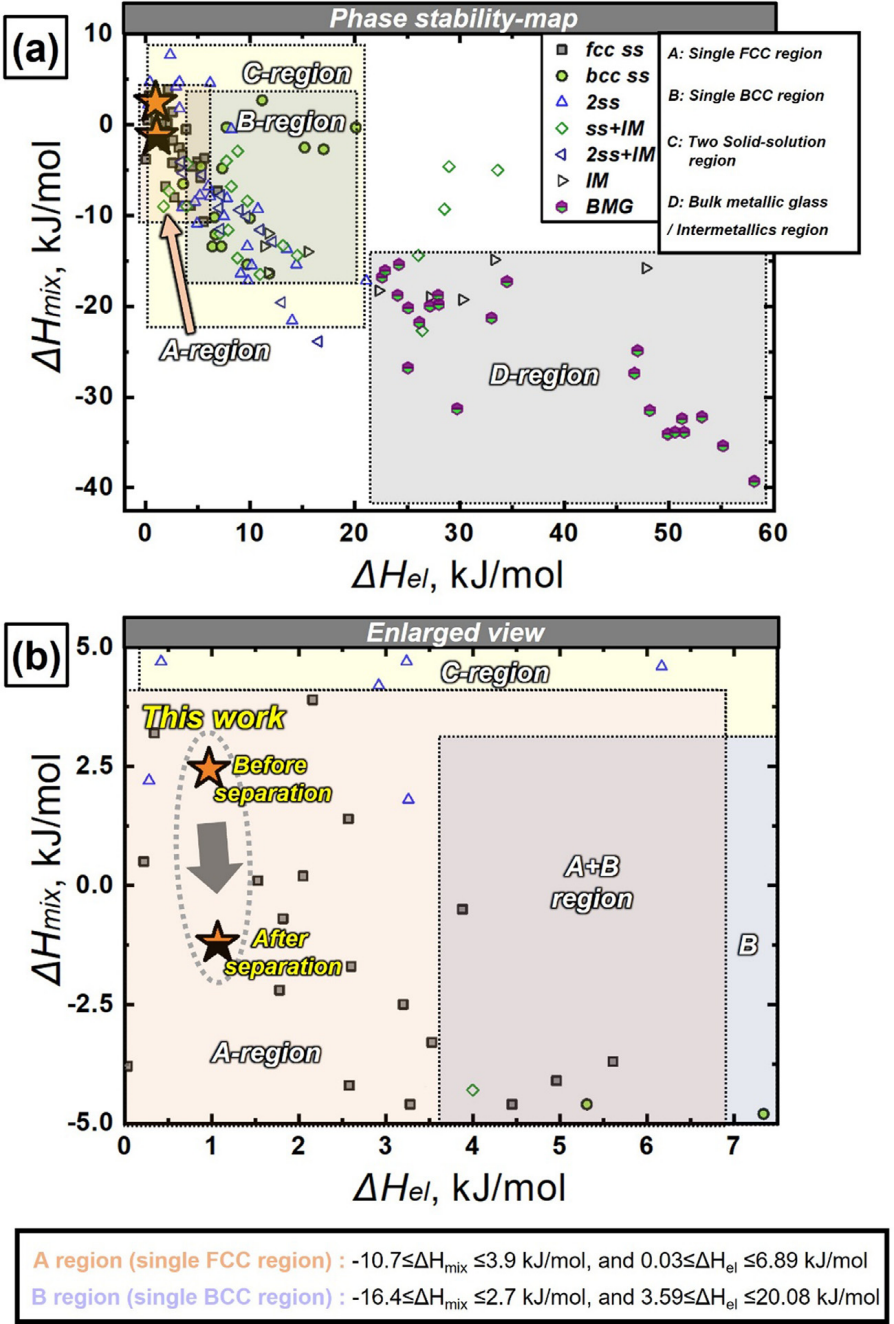
(a) Enthalpy of mixing (ΔH_mix_) vs. lattice strain energy (ΔH_el_) criterion used for the prediction of stable phases in CoCrCu_1.5_MnNi in HEAs obtained from Ref. [4], and (b) Enlarged view of inset rectangle marked in (a).

**Fig. 3:**
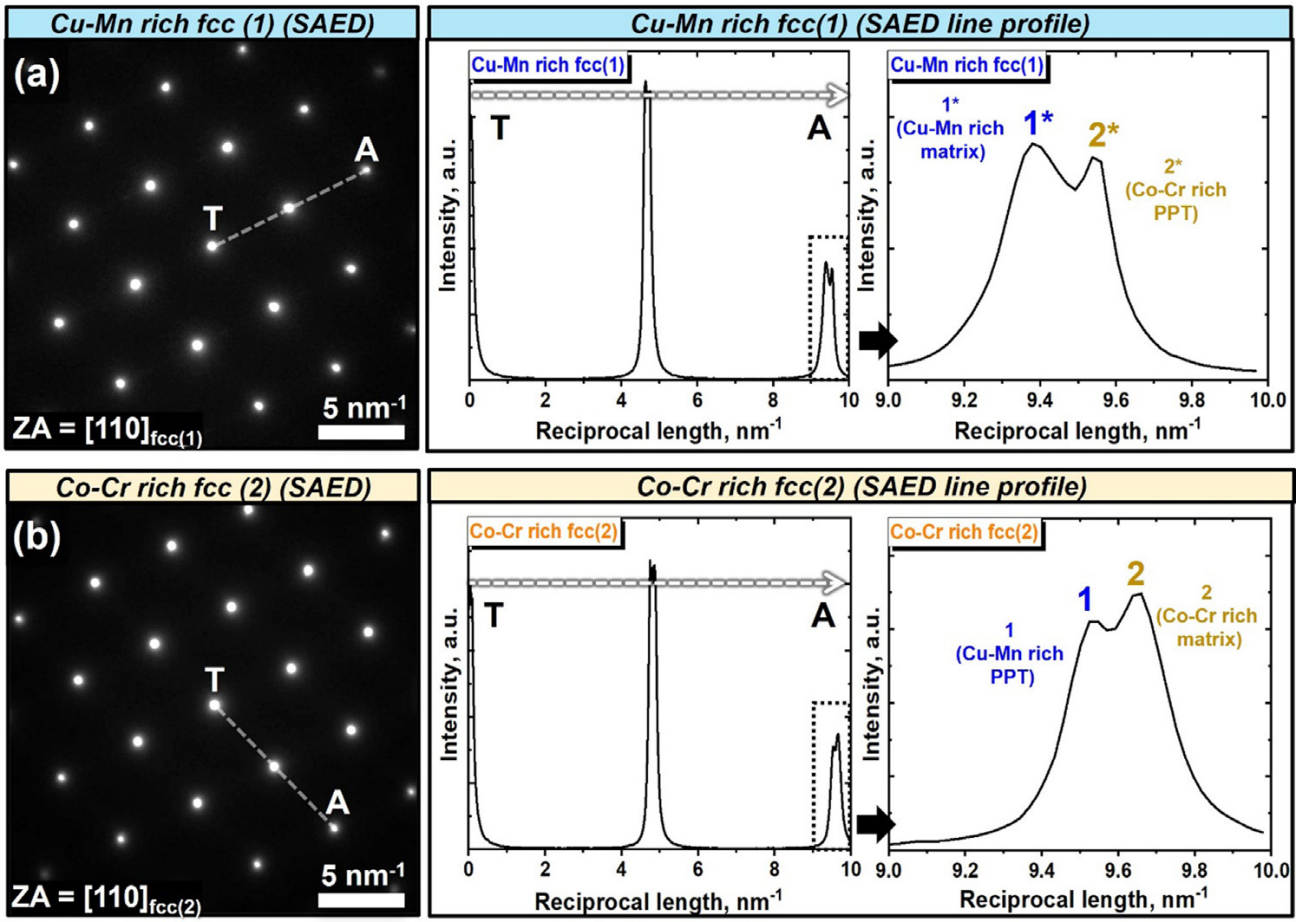
Selected area diffraction patterns and the intensity distribution line profile from the (a) Cu-Mn rich fcc(1), and (b) Co-Cr rich fcc(2) regions. Line profile obtained the electron diffraction spots along the dotted line from the center (T) to one of the (111) reflections.

Fig. 2 shows the enthalpy of mixing (ΔH_mix_) versus lattice strain energy (ΔH_el_) criterion used for the prediction of stable phase region [4,5]. Fig. 2(a) exhibits the phase stability space map based on ΔH_mix_ and ΔH_el_ criterion using the various HEA data gathered from the literature [4]. The calculated values of ΔH_mix_ and ΔH_el_ for various HEAs are stored in Mendeley Data repository. The four different regions in the (ΔH_mix_) versus (ΔH_el_) space exhibits the regions identified as single fcc, single bcc, 2 solid solution, and bulk metallic alloy regions. The HEAs with stable fcc structure (designated as “A region”) were found to be -10.7< ΔH_mix_<3.9 kJ/mol, and 0.03 kJ/mol < ΔH_el_<6.89 kJ/mol. Herein, calculated values of the as-cast CoCrCu_1.5_MnNi before/after phase separation, which are represented by red star symbols, in Fig. 2(b) from the inset of Fig. 2(a) were placed on the stable fcc and 2ss regions, (Table 3). Fig. 3 exhibits the selected area diffraction patterns from the (a) Cu-Mn rich fcc (1) and (b) Co-Cr rich fcc (2) phase including both the matrix and precipitates and the intensity distribution line profile of electron diffraction spots along the dotted line from the center (T) to one of the (111) reflections (obtained from Figs. 2(a) and 3(a) in Ref. [1]). The intensity distribution line profiles of electron diffraction spots were presented in Mendeley Data repository (“Fig.3-SAED line scan.xlsx”). Fig. 3(a) shows the SAED pattern and line scan profile obtained from the electron diffraction spots along the dotted line from the center (T) to one (A) of the (111) reflections in Cu-Mn rich fcc (1) phase. In the same way, the SAED pattern and line scan profile were obtained from the electron diffraction spots in Co-Cr rich fcc(2) phase, presented in Fig. 3(b). It should be noted that the separation at the tip of the (111) reflection peaks occurred. The degree of diffraction peak separation from dual fcc phase structure may depend on the specimen interaction volume of diffraction, composition and the atomic size difference between constituent elements. Selected area electron diffraction patterns in Fig. 3 were taken from a smaller volume of either granular fcc(1) (Fig. 3(a)) or fcc(2) (Fig. 3(b)). Diffraction spots in Fig. 3(a) and (b) were from both the matrix and the precipitates in each Cu-Mn interdendritic and Co-Cr dendritic region. Since the lattice of precipitates were influenced or constrained by the matrix, the diffraction spots from the matrix and precipitates with the same crystalline structure appear to be merged [5], [6], [7].

The tip separation of all designated (111) reflection from both Cu-Mn rich fcc(1) and Co-Cr rich fcc(2) phases should be noted. The peak separation is attributed to the dual fcc phase structure, i.e, the fcc matrix and the fcc precipitates. Because of the larger atomic radius of Cu compared to those of other constituent elements, the left peak from the tip shoulder is thought to be Cu-Mn rich matrix (designated as “1*”) and right peak is the Co-Cr rich precipitates (designated as “2*”) in Fig. 3(a). Likewise, left peak and right peak in Fig. 3(b) is supposedly from Cu-Mn rich precipitates (designated as “1”)) and Co-Cr rich matrix (designated as “2”)), respectively. The converted lattice constants from these diffraction tip from both matrix and precipitates are around to be, a_1*_=0.3691 nm, a_2*_=0.3630 nm and a_1_=0.3632 nm, a_2_= 0.3589 nm, respectively. Their differences from the matrix and precipitates are similar to the lattice constant from the Cu-Mn rich fcc(1) interdendrite phase (0.3670 nm) and Co-Cr rich fcc(2) dendrite phase (0.3601 nm) obtained from the XRD of Fig. 1(a) in Ref. [1]. The calculation of lattice parameters from the XRD is considered to be more accurate than that from the TEM SAED [7]. The expanded view in Fig. 3 showed the presence of peak separation from the matrix and the precipitates.

**Fig. 4:**
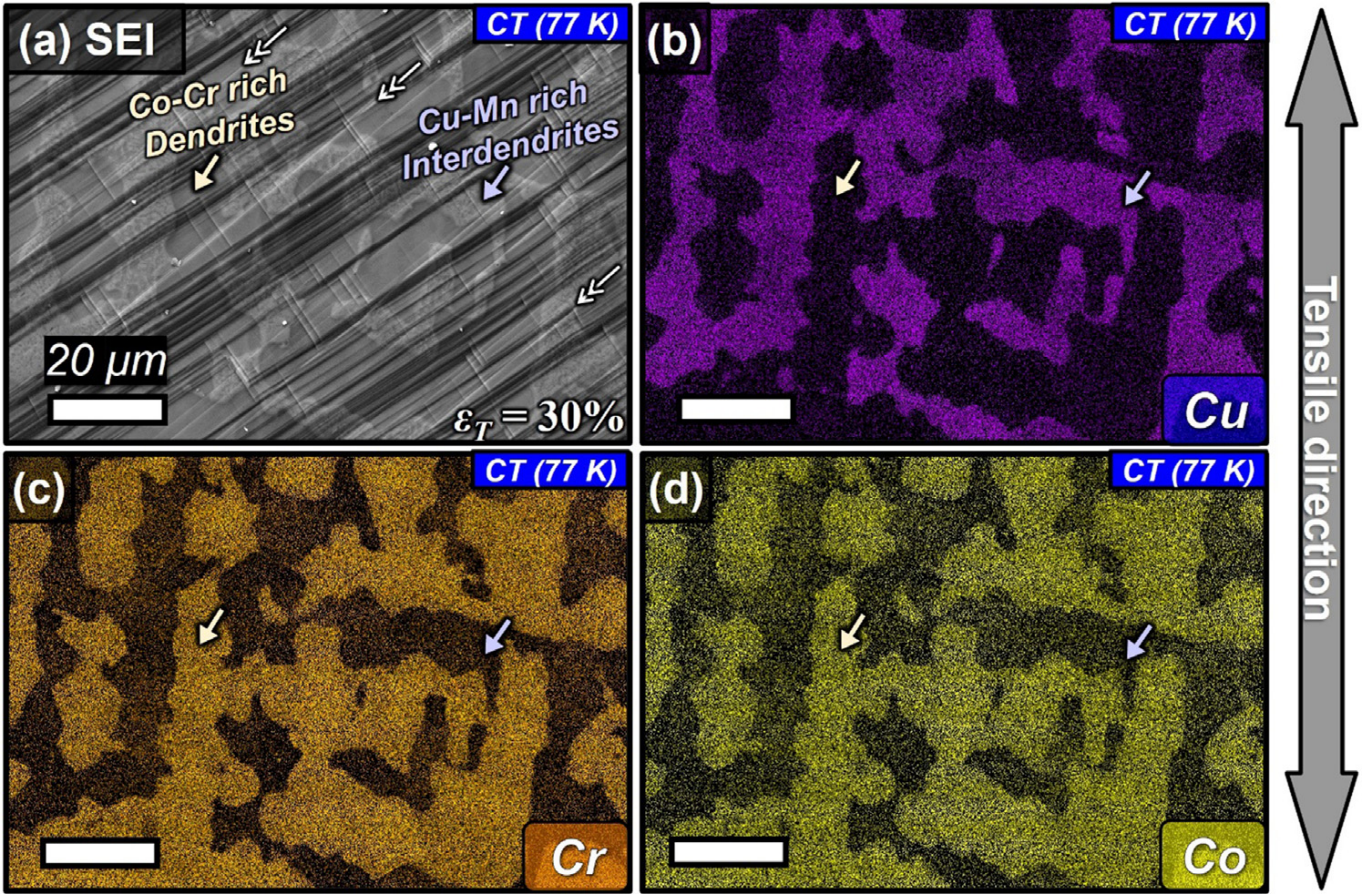
Surface slip morphologies developed in the dual fcc phase structure of CoCrCu_1.5_MnNi after 30% strain at 77 K, the secondary electron image (a), and EDS mapping images (b-d) of Cu (b), Cr (c), and Co (d), respectively.

**Fig. 5:**
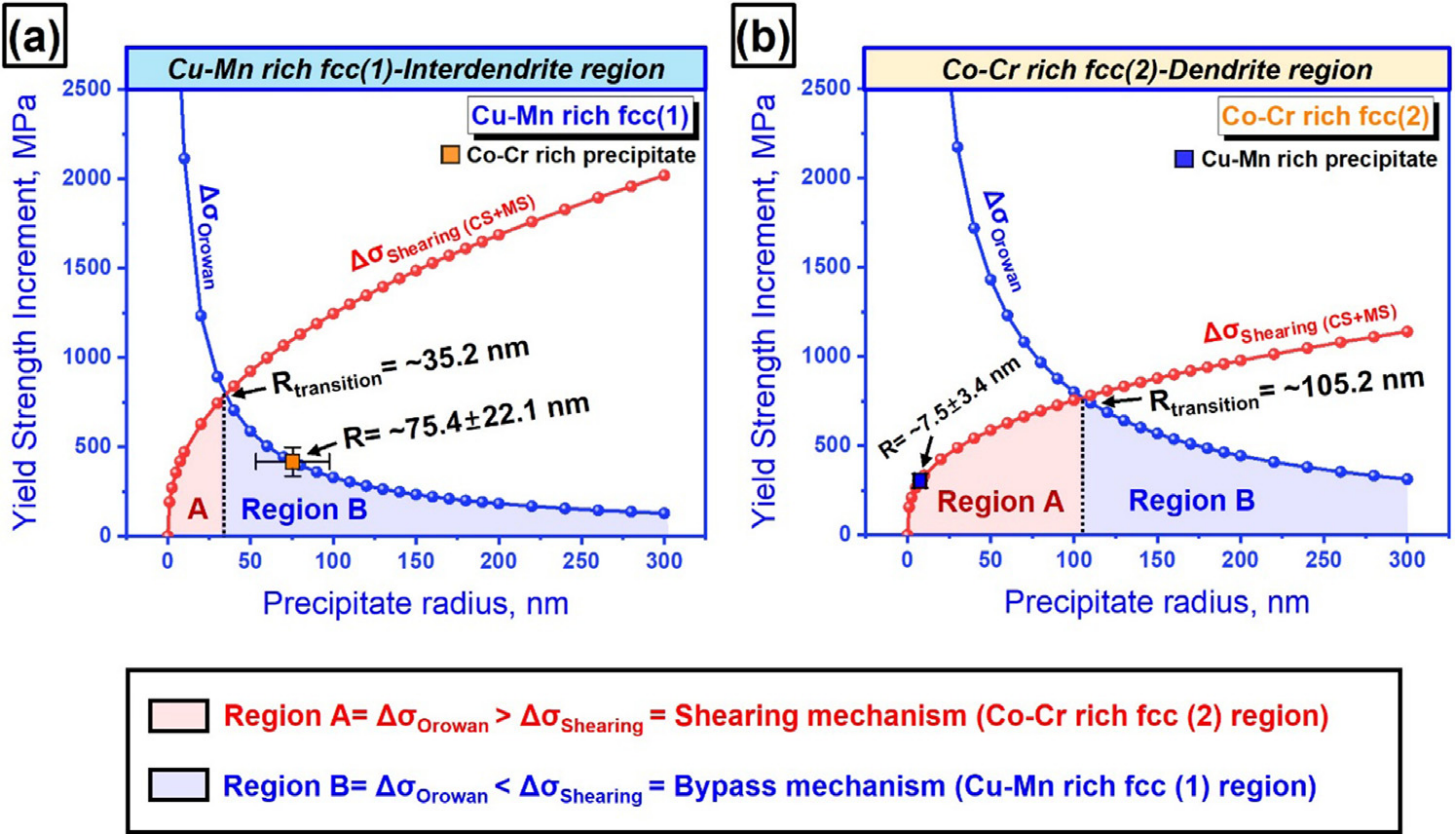
Precipitation strengthening at cryogenic temperature (a, b) by shearing mechanism and Orowan bypassing strengthening as a function of radius of needle-shaped precipitates in Cu-Mn rich inter-dendritic phase (a) and Co-Cr dendritic phase (b).

Fig. 4 shows the surface slip morphologies developed in the dual fcc phase structure of CoCrCu_1.5_MnNi after 30% strain at 77 K, the secondary electron image (a), and EDS mapping images (b-d) of Cu (b), Cr (c), and Co (d), respectively. Bright region is the Cu-Mn rich interdendritic region with Co-Cr precipitates (b) and the dark region is the Co-Cr rich dendritic region with Cu-Mn precipitates. In Fig. 5, the precipitation strengthening at cryogenic temperature (a, b) by shearing mechanism and Orowan bypassing strengthening as a function of radius of needle-shaped precipitates in Cu-Mn rich fcc(1) inter-dendritic phase (a) and Co-Cr fcc(2) dendritic phase (b), were presented. The calculated values of the precipitation strengthening either from the Orowan strengthening or the precipitate shear strengthening in Fig.5 are stored in Mendeley Data repository. The comparison of the critical radius in Fig. 11 of Ref.1 with that shown in Fig. 5 supports that the small increase of the shear modulus at 77 K does not have a significant influence the dominant precipitation strengthening.

**Table 1:** The measured phase fraction, relative phase fraction of each matrix and precipitate, radius of the precipitates, and chemical composition of dual fcc structure and embedded precipitates from their matrices in as-cast CoCrCu_1.5_MnNi determined by XRD, SEM, TEM and STEM-EDS analysis.

					Chemical composition (at%)				

Regions	Phase	Phase fraction (%)	Relative phase fraction (%)	Particle Radius (nm)	Cr	Mn	Co	Ni	Cu
Cu-Mn rich fcc(1) Interdendritic regions	Cu-Mn rich matrix	45.6	80.5	-	2.0	29.0	4.9	23.2	41.0
	Co-Cr rich precipitates		19.5	75.4	29.6	19.0	29.8	18.9	2.8

Co-Cr rich fcc(2) Dendritic regions	Co-Cr rich matrix	54.4	64.8	-	27.0	19.1	27.3	22.3	4.3
	Cu-Mn rich precipitates		35.2	7.5	18.2	20.6	17.8	22.1	21.4

Table 1 summarizes the measured phase fraction of the microscale separated phases, relative phase fraction of each matrix and precipitate in each microscale phase, radius of the precipitates, and chemical composition of dual fcc structure and embedded precipitates from their matrices in as-cast CoCrCu_1.5_MnNi determined by XRD, SEM, TEM and STEM-EDS analysis. In Table 2, the lattice constants of dual fcc phases and their precipitates were calculated using the atomic radius of each constituent element based on the composition of the phases obtained from STEM-EDS from Table 1. In Table 3, the calculated ΔH_mix_ and ΔH_el_ values of before and after phase separation in the as-cast non-equiatomic CoCrCr_1.5_MnNi, and equiatomic CoCrCuMnNi HEAs were presented. It shows the energy reduction after phase separation in as-cast non-equiatomic CoCrCr_1.5_MnNi (-3.55 kJ/mol) is greater than that in as-cast equiatomic CoCrCuMnNi (-1.52 kJ/mol), supporting the more pronounced phase separation/precipitation in the non-equiatomic CoCrCr_1.5_MnNi.

**Table 2:** The lattice constants of dual fcc phases and their precipitates were calculated using the atomic radius [7] of each constituent element based on the composition of the phases obtained from STEM-EDS.

Regions	Phase	Calculated lattice constant (nm)
Cu-Mn rich fcc(1) Interdendritic regions	Cu-Mn rich matrix	0.3582
	Co-Cr rich precipitates	0.3568

Co-Cr rich fcc(2) Dendritic regions	Co-Cr rich matrix	0.3568
	Cu-Mn rich precipitates	0.3573

**Table 3:** Calculated enthalpy of mixing (ΔHmix) and lattice distortion energy (ΔHel), before (Δ$H_{\text{mix}/\text{el}}^{\text{single}}$) and after separation (Δ$H_{\text{mix}/\text{el}}^{\text{dual}}$) into dual fcc phases. Energy reduction in mixing enthalpy (Δ$H_{\text{mix}}^{\text{reduction}}$), lattice distortion energy (Δ$H_{\text{el}}^{\text{reduction}}$), and total chemical enthalpy (Δ$H_{\text{tot}}^{\text{reduction}}$) after separation are calculated in non-equiatomic CoCrCu1.5MnNi and equiatomic CoCrCuMnNi HEAs.

						Δ$H_{\text{mix}}^{\text{reduction}}$, (kJ/mol)	Δ$H_{\text{el}}^{\text{reduction}}$, (kJ/mol)	Δ$H_{\text{tot}}^{\text{reduction}}$, (kJ/mol)

Alloy	Phase	ΔH_mix_, (kJ/mol)		ΔH_el_, (kJ/mol)		[Δ$H_{\text{mix}}^{\text{dual}}$-Δ$H_{\text{mix}}^{\text{single}}$]	[Δ$H_{\text{el}}^{\text{dual}}$-Δ$H_{\text{el}}^{\text{single}}$]	[Δ$H_{\text{mix}}^{\text{reduction}}$+Δ$H_{\text{el}}^{\text{reduction}}$]
Non-equiatomic CrMnCoNiCu_1.5_ (This work)	Single phase (Before separation)	2.44, (Δ$H_{\text{mix}}^{\text{single}})$		0.97, (Δ$H_{\text{el}}^{\text{single}})$		-3.65	0.1	-3.55
	Cu-Mn rich fcc(1) (After separation)	2.78	Average -1.21, (Δ$H_{\text{mix}}^{\text{dual}})$	0.90	Average 1.07, (Δ$H_{\text{el}}^{\text{dual}}$)			
	Co-Cr rich fcc(2) (After separation)	-4.55		1.22				

Equiatomic CrMnCoNiCu	Single phase (Before separation)	0.64, (Δ$H_{\text{mix}}^{\text{single}})$		1.05, (Δ$H_{\text{el}}^{\text{single}})$		-1.54	-0.02	-1.52
	Cu-Mn rich fcc(1) (After separation)	2.12	Average -0.90, (Δ$H_{\text{mix}}^{\text{dual}})$	0.97	Average 1.07, (Δ$H_{\text{el}}^{\text{dual}}$)			
	Co-Cr rich fcc(2) (After separation)	-2.91		1.14				

## 2 Experimental Design, Materials and Methods

Non-equimolar CoCrCr_1.5_MnNi ingots were cast by vacuum induction melting (VIM) under a high-purity Ar atmosphere. Phase structure analyses of as-cast microstructure were carried out employing X-ray diffraction (XRD), field-emission scanning-electron-microscope (FE-SEM) equipped with energy dispersive spectroscopy (EDS) detectors. Mechanical polishing method for deformed microstructure of SEM and EDS analysis was carried out down to 1μm using SiC papers and diamond suspension. Then, final preparation was done by auto-polishers (MetPrep 3TM, Allied High-Tech Products Inc.) with colloidal silica including a size of particle of 0.04 μm around 4h. After that, the tensile specimens with mirror-like plane were tested at 30% strain at cryogenic temperature. Transmission-electron microscopy (TEM) analyses were performed using JEOL JEM-2100F operated at an acceleration voltage of 200 kV. TEM foils of as-cast CoCrCu_1.5_MnNi were prepared using a dimple grinder (Gatan, model 656, USA) and the precision ion polishing system (Gatan, model 691, USA), with argon gas ions at 3.6 keV. To study the effect of ductility on the gage length to gage width ratio in as-cast CoCrCr_1.5_MnNi, the dogbone-type tensile specimens with the gage dimension of 9 × 3.4 × 1.0 mm^3^ and 13.6 × 3.4 × 1.0 mm^3^ were machined by electro-discharging machine (EDM). Uniaxial tensile testing was performed with the strain-rate of 10^–3^ s^–1^, at both room and cryogenic temperatures.

## Ethics Statements

The authors followed universally expected standards for ethical behavior in conducting and publishing scientific research.

## CRediT authorship contribution statement

**Sang Hun Shim:** Formal analysis, Data curation, Investigation, Writing – original draft. **Hesam Pouraliakbar:** Formal analysis, Data curation, Investigation, Writing – original draft. **Byung Ju Lee:** Formal analysis, Data curation, Investigation. **Yong Keun Kim:** Formal analysis, Data curation. **Mohsen Saboktakin Rizi:** Formal analysis, Data curation. **Jun Hyun Han:** Methodology, Data curation, Writing – review & editing. **Sun Ig Hong:** Conceptualization, Supervision.

## Declaration of Competing Interest

The authors declare that they have no known competing financial interests or personal relationships that could have appeared to influence the work reported in this paper.

## Data Availability

Supporting data for strengthening and deformation behavior of as-cast CoCrCu1.5MnNi high entropy alloy with micro-/nanoscale precipitation (Original data) (Mendeley Data).
